# How Interest in Phages Has Bloomed into a Leading Medical Research Activity in Poland

**DOI:** 10.3390/v14122617

**Published:** 2022-11-24

**Authors:** Maciej Żaczek, Andrzej Górski, Andrzej Grzybowski, Edyta Pasternak, Beata Weber-Dąbrowska, Ryszard Międzybrodzki

**Affiliations:** 1 Bacteriophage Laboratory, Ludwik Hirszfeld Institute of Immunology and Experimental Therapy, Polish Academy of Sciences, 53-114 Wrocław, Poland; 2 Phage Therapy Unit, Ludwik Hirszfeld Institute of Immunology and Experimental Therapy, Polish Academy of Sciences, 53-114 Wrocław, Poland; 3 Infant Jesus Hospital, Medical University of Warsaw, 02-005 Warsaw, Poland; 4 Institute for Research in Ophthalmology, 60-836 Poznań, Poland; 5 Department of Ophthalmology, University of Warmia and Mazury, 10-561 Olsztyn, Poland; 6 Department of Clinical Immunology, Transplantation Institute, Medical University of Warsaw, 02-006 Warsaw, Poland

**Keywords:** phage therapy, medical sciences, ranking

## Abstract

Poland has a leading position in phage therapy, as reflected by the number of patients treated and relevant publications in quality journals. The Institute of Immunology and Experimental Therapy of the Polish Academy of Sciences was established by Ludwik Hirszfeld, a prominent microbiologist and serologist who also initiated studies on phages and pioneered the activities that set into motion phage therapy at the Institute. To achieve this goal, Hirszfeld had to overcome many difficulties in a post-war Poland. He died a month before the official start of the Institute’s activity and was not able to witness the advancement of the Institute bearing his name. However, his hard work and dedication have been recently rewarded. In a recent evaluation of scientific performance, the Institute received the highest ranking in medical sciences among all universities and research institutions in Poland. One could consider it a posthumous tribute to the memory of L. Hirszfeld, being well-deserved on the grounds of the Institute’s achievements (especially in the field of phage therapy) as well as his life and work.

## 1. Introduction

The creation of the Hirszfeld Institute of Immunology and Experimental Therapy of the Polish Academy of Sciences in Wrocław, Poland, is closely related with the final chapter of Ludwik Hirszfeld’s life. In fact, in his memoir, entitled “Ludwik Hirszfeld: The Story of One Life”, the name Wrocław, a city that was returned to Polish borders after World War II, does not appear until the very end [1]. Ludwik Hirszfeld was one of the most prominent microbiologists and serologists of the 20th century, being particularly engaged in promoting medical ethics and the principles of human dignity. Hirszfeld was also the founder and first director of the Institute [1,2]. In this article, we want to focus on the period prior to the establishment of the Institute and the role of Ludwik Hirszfeld in this process, which we believe has never been discussed before in the scientific literature.

## 2. Ludwik Hirszfeld’s First Years in Wrocław

Prof. Ludwik Hirszfeld came to Wrocław shortly after the end of the war on 1 August 1945. He brought his phage collection with him, which had previously been stored in the State Department of Hygiene in Warsaw [3]. This constituted the very beginning of phage research and therapy in Poland; 50 years later, Wrocław remains an active center of phage research, with the Wrocław center becoming the first treatment facility in the European Union and serving as a database for the largest group of patients that have undergone phage therapy worldwide, operating in accordance with current legal and ethical requirements. However, Hirszfeld did not move to Wrocław because of phages. Back then, he was entrusted with quite a different task. The authorities appointed him to the position of an organizer of the Faculty of Medicine at the University of Wrocław, a mission that he himself described as being one of the most difficult times in his life. Most of the university facilities were severely damaged as a result of hostilities, posing a multitude of organizational challenges described in detail in his memoir [1]. Hirszfeld began this role with one assistant who had an academic background and one student [4]. However, his hard work and devotion soon began to bear fruit. In 1946, his department had approximately 100 employees. By analogy, the student population in Wrocław skyrocketed just one year after the end of WWII and constituted 160% of students learning at Wrocław universities before the war. Organizational and administrative matters so absorbed him that he soon started neglecting his research work. In 1946, thanks to his international contacts, he was able to visit the US together with his wife. During this time, he also visited Prof. James Craigie in Toronto and was impressed by his work on phages, which he described as “small creatures that attack bacteria” [5]. Hirszfeld’s fascination continued to grow after his return to Poland. In 1948, Hirszfeld implemented a phage application for typing bacterial strains, a method that has become universal and is still used today around the globe [3]. During this time, Hirszfeld referred to phage research in many of his publications and lectures, as well as in his private correspondence, believing that this phenomenon should be further explored [5].

Hirszfeld’s abovementioned devotion to science is clearly visible in a letter dated 8 July 1952 (five months before the official establishment of the Institute) and addressed to a Polish communist activist, Ostap Dłuski [6]. The letter concerned the potential participation of Hirszfeld in the official delegation to Korea as part of the so-called “Korean Commission” investigating global biohazards. Hirszfeld did not accept the invitation. This decision was motivated by, among other things, the intensive research work carried out at the Department of Medical Microbiology, which was soon to be transformed into an Institute. He led eight research topics that required, according to him, “constant supervision and reporting”. Despite his advanced age and physical disabilities, Hirszfeld described himself as an “irreplaceable person”. Undoubtedly, the role of scientist suited him best, and this aspect of his life was more important than politics and international recognition. Hirszfeld made sure that the research conducted at the Department of Medical Microbiology was related to the health problems of the Poles, a nation that suffered tremendous damage as a result of the war. He implemented the theory of serological conflict in practice, saving the life of hundreds of children. His student, Feliks Milgrom, organized large-scale activities for diagnosing sexually transmitted diseases [7]. These activities included over half a million people and identified several thousands of cases of syphilis [4]. In addition, the Polish blood donation sector was reorganized, which included the determination of newly discovered blood groups and the training of laboratory workers dealing with blood donation. Under the leadership of Hirszfeld, the Department of Medical Microbiology developed scientific activity in the field of medical microbiology, immunology and biochemistry, established research collaborations with numerous clinics and quickly trained new assistants. From 1951 to 1952, the department was expanded. A special didactic building was created to house a lecture hall with 250 seats and a practice room with 100 seats. New facilities for outpatient clinics, administration, and a student reading room were also built. Additionally, the old department building underwent a major renovation and was equipped with the newest instruments at that time, such as an electrophoresis apparatus, an ultracentrifuge and a spectrophotometer [4]. Soon, all these buildings and equipment would be taken over by the newly established Institute. However, being one of scientists who rejected the proposal to join the communist party, Hirszfeld’s efforts to establish the Institute were met with prolonged resistance from the authorities [8]. The new dogma of genetic sciences gaining ground in the Soviet Union, which was opposed by international scientific authorities such as Hirszfeld, certainly did not help the cause.

## 3. The Beginnings of the Hirszfeld Institute

Despite these obstacles, on 2 December 1952, after many years of efforts, pursuant to Resolution No. 70 of the Polish Academy of Sciences, the Department of Medical Microbiology in Wrocław was transformed into the Institute of Immunology and Experimental Therapy [5,9]. According to the Government Presidium, the two main objectives of the Institute were to conduct research in the field of immunology and experimental therapy—in accordance with the plan approved by the Presidium of the Polish Academy of Sciences—and to conduct teaching activities in the field of microbiology—in accordance with the applicable educational program [10]. Further provisions stated that all employees of the Department of Medical Microbiology would be acquired by the Institute along with all the facilities and equipment mentioned above. The Institute acquired legal personality on the basis of a resolution of the Presidium of the Polish Government established on 6 February 1954, just one month before Hirszfeld’s death. In recognition of his contributions to Polish and international science and the large part he played in its organization, the Institute was named after its founder, Ludwik Hirszfeld. Upon obtaining the legal status of an independent research institution, the Institute had 89 employees, including 32 research employees (1 professor and 2 associate professors), 20 scientific and technical employees, 13 administration employees and 23 service workers [11]. The second and longest-serving Director of the Hirszfeld Institute, Prof. Stefan Ślopek, during a lecture given at the Presidium of the Polish Academy of Sciences on 23 May 1955, emphasized the irreparable loss caused by Hirszfeld’s death, who passed away on the eve of the initiation of the Institute. In Ślopek’s own words, “(…) *in this most difficult, initial period of the Institute’s development, suddenly there was no man with outstanding organizational talent and great authority*” [12].

The crisis in which the Institute found itself was overcome a year later. In early 1955, several professors of the Medical Academy in Wrocław expressed their readiness to cooperate with the Institute, taking over the management of departments or laboratories and strengthening the Institute’s scientific staff. After only five years of the Institute’s activity, the number of employees had almost doubled, and in the following years, it systematically increased. The research plan for 1955 included seven research fields, including experimental therapy, which covered phage therapy. Experimental therapy was also included in more distant research plans, including the years 1956–1960, which was a clear signal that the Hirszfeld Institute had become a permanent home for research on phages [12]. After 20 years, in the first year of operation in the new facilities built for the needs of the Institute (the lack of which was brought to authorities’ attention by Ślopek during the aforementioned lecture), the Institute employed 419 people, and in the years 1956–1974, the Scientific Council of the Institute awarded 165 academic degrees [11]. As for phage therapy, more than 1000 patients received treatment in the years 1954–1987 [13]. The breadth of research undertaken by the Institute encompasses biology and medical sciences and includes medical microbiology and glycobiology, microbial immunochemistry and vaccines, tumor immunology, microbiome immunobiology, biology of stem and neoplastic cells, immunogenetics and clinical immunology and experimental anticancer therapy.

## 4. The Promotion of the Institute with Progress in Phage Research and Therapy

Although Hirszfeld was clearly fascinated by phages, a sentiment he expressed during a lecture in 1948 at a meeting of the Wrocław Scientific Society entitled “*Walka świata niewidzialnego z pozawidzialnym*” (The battle of the invisible with the imperceptible) [14], in his three-page curriculum vitae dated 20 May 1952, Hirszfeld did not mention the phages even once [15]. One can assume that in a document summarizing his scientific journey, he focused on his internationally recognized achievements, leaving this area of interest to other, more experienced phage scientists. This approach corresponds with his high ethical standards, modesty and respect for others, as he so often mentioned among his colleagues, associates and students [16]. However, he might not have pursued his interest in phages had history played out differently. Certainly, there would not be a Phage Therapy Unit as we know it today without Hirszfeld’s efforts to initiate the Institute of Immunology and Experimental Therapy of the Polish Academy of Sciences. At the meeting held the day after Ludwik Hirszfeld’s funeral, a team of Institute employees, based on a careful analysis of the situation at the Institute, stated that all scientific work would be continued. The team strove to honor Hirszfeld’s memory through intense and continuous research [4]. One such example is the multidisciplinary scientific journal *Archivum Immunologiae et Therapiae Experimentalis*, founded by Hirszfeld in 1953, which is continuously published on behalf of the Hirszfeld Institute to this day. The journal is indexed in the major international scientific databases (including Medline), and in 2016 the editorial team of the journal (A. Górski, H. Krotkiewski, M. Zimecki, A. Steć) received a scientific award from the President of Polish Academy of Sciences for earning notable international prestige for the journal due to its high publishing standards over many years of publication.

In our two most recent articles, we presented how phage therapy in Poland has made “a centennial journey to the first ethically approved treatment facility in Europe” [5] as well as “its history, milestones and international recognition” [13]. This journey has led our Institute to the most prominent position among all universities and research institutions in Poland, as indicated by its acquisition of the highest scientific score in medical sciences in line with its ranking by the Ministry of Education and Science (MEiN).

Every four years, the MEiN evaluates the output of Polish universities and research institutions in all scientific disciplines. For this purpose, publications, grants, patents and the so-called social influence of research are assessed. The results of the most recent evaluation were disclosed in July 2022. As already mentioned, our Institute has been awarded the highest ranking in medical sciences (A+). Given the number of articles published by our team, the work on phage therapy contributed greatly to this rating.

Of course, each evaluation of research activity may be criticized, and the present one is no exception. However, this result is well-supported by our publications, patents, international recognition and novel theories on phage repurposing. Furthermore, the ethical aspects of our work also deserve attention; our patents are owned by the Institute, and no member of our phage therapy team, led by Prof. A. Górski, has any ties to industry or other commercial entities. Moreover, our Institute does not financially profit from these therapy activities; conversely, each year it provides funding covering some of the financial deficit of our unit. In other words, significant progress in medical research is possible without commercial ties with industry—despite those who insist that such a relationship is a pre-condition for success.

In an article dedicated to L. Hirszfeld, it is noted that “Hirszfeld’s role in introducing research on bacteriophages and phage therapy in Poland has also gone wholly unrecognized. This is all the more striking as publications and other documents attest to this, as well as to his bringing phages to our Institute from the National Institute of Hygiene in Warsaw.” In his “A Story of One Life”, Hirszfeld recalls his group’s research on bacteriophages. One of his assistants at the National Institute of Hygiene “has published a valuable paper about bacteriophages,” and a second “has won herself an international name thanks to a beautiful paper on bacteriophages” (Czytelnik 2000, p. 399). In fact, there is no doubt that it was Hirszfeld who began the work which resulted in the unique position that our country and Institute have attained in this field. He underscored in these, for example, that the potency of phages’ antibacterial effect is greater than the strength of the organism’s resistance. In 2004, the year in which we paid tribute to the memory of Ludwik Hirszfeld on the 50th anniversary of his death, we formulated a hypothesis suggesting that endogenic bacteriophages (those present in our bodies) play a significant role in immune regulation, thus fulfilling functions that have previously been ascribed exclusively to the immune system [17,18].

## 5. Final Remarks

We hope that the success of research on phage therapy extends well beyond the limits of this therapy to applications in other medical disciplines. Recent progress in phage immunobiology may enable phage therapy application beyond anti-bacterial action (phage repurposing), including in gastroenterology, transplantation, viral diseases, etc. [19]. We also hope that other examples will follow. This could perhaps influence the current trend of the total commercialization of medicine at the expense of public interest.
